# Clinical validation of a 90-gene expression test for tumor tissue of origin diagnosis: a large-scale multicenter study of 1417 patients

**DOI:** 10.1186/s12967-022-03318-6

**Published:** 2022-03-07

**Authors:** Wei Sun, Wei Wu, Qifeng Wang, Qian Yao, Qin Feng, Yue Wang, Yu Sun, Yunying Liu, Qian Lai, Gu Zhang, Peng Qi, Yifeng Sun, Chenhui Qian, Wanli Ren, Zhengzhi Luo, Jinying Chen, Hongying Wang, Qinghua Xu, Xiaoyan Zhou, Wenyong Sun, Dongmei Lin

**Affiliations:** 1 Department of Pathology, Key Laboratory of Carcinogenesis and Translational Research (Ministry of Education), Peking University Cancer Hospital & Institute, No.52 Fucheng Road, Wu Ke Song, Haidian District, Beijing, China; 2 Department of Pathology, Cancer Hospital of the University of Chinese Academy of Sciences (Zhejiang Cancer Hospital), No.1 East Road of Banshan, Hangzhou, Zhejiang China; 3 Department of Pathology, Fudan University Shanghai Cancer Center, No.270 Dong’An Road, Shanghai, China; 4 Department of Oncology, Shanghai Medical College, Fudan University, Shanghai, China; 5 Institute of Pathology, Fudan University, Shanghai, China; 6 The Cancer of Unknown Primary Group of Pathology Committee, Chinese Research Hospital Association, Shanghai, China; 7 The Canhelp Genomics Research Center, Canhelp Genomics Co., Ltd., Hangzhou, China; 8 The Institute of Machine Learning and Systems Biology, College of Electronics and Information Engineering, Tongji University, Shanghai, China; 9 Xuzhou Engineering Research Center of Medical Genetics and Transformation, Department of Genetics, Xuzhou Medical University, Xuzhou, China

**Keywords:** Tissue of origin, Gene expression profiling, Real-time PCR, Tumor classification, The 90-gene expression assay

## Abstract

**Background:**

Once malignancy tumors were diagnosed, the determination of tissue origin and tumor type is critical for clinical management. Although the significant advance in imaging techniques and histopathological approaches, the diagnosis remains challenging in patients with metastatic and poorly differentiated or undifferentiated tumors. Gene expression profiling has been demonstrated the ability to classify multiple tumor types. The present study aims to assess the performance of a 90-gene expression test for tumor classification (i.e. the determination of tumor tissue of origin) in real clinical settings.

**Methods:**

Formalin-fixed paraffin-embedded samples and associated clinicopathologic information were collected from three cancer centers between January 2016 and January 2021. A total of 1417 specimens that met quality control criteria (RNA quality, tumor cell content ≥ 60% and so on) were analyzed by the 90-gene expression test to identify the tumor tissue of origin. The performance was evaluated by comparing the test results with histopathological diagnosis.

**Results:**

The 1417 samples represent 21 main tumor types classified by common tissue origins and anatomic sites. Overall, the 90-gene expression test reached an accuracy of 94.4% (1338/1417, 95% CI: 0.93 to 0.96). Among different tumor types, sensitivities were ranged from 74.2% (head&neck tumor) to 100% (adrenal carcinoma, mesothelioma, and prostate cancer). Sensitivities for the most prevalent cancers of lung, breast, colorectum, and gastroesophagus are 95.0%, 98.4%, 93.9%, and 90.6%, respectively. Moreover, specificities for all 21 tumor types are greater than 99%.

**Conclusions:**

These findings showed robust performance of the 90-gene expression test for identifying the tumor tissue of origin and support the use of molecular testing as an adjunct to tumor classification, especially to those poorly differentiated or undifferentiated tumors in clinical practice.

**Supplementary Information:**

The online version contains supplementary material available at 10.1186/s12967-022-03318-6.

## Introduction

The cancer burden is rising rapidly due to the aging of the population and the adoption of unhealthy lifestyle behaviors, which became the leading cause of death in China [1]. Once malignancy tumors were diagnosed, the determination of tissue origin and tumor type is critical for clinical management. In routine clinical practice, tumor diagnosis requires a comprehensive synthesis of the clinical and pathological findings. At present, although the significant advance in imaging techniques and histopathological approaches, including morphology and immunohistochemistry (IHC), the diagnosis remains challenging in patients, which initially presenting with metastatic and poorly differentiated or undifferentiated tumors [2–5].

In the past decade, different approaches based on gene expression profiling, DNA methylation, and genomic alteration were developed to identify tumor tissue of origin [6–8]. Many of these assays compared the molecular profiles of the test sample as determined by either microarray, next-generation sequencing (NGS), or real-time PCR (RT-PCR) to molecular profiles of tumors with confirmed tumor types. Two commercialized assays termed Tissue of Origin (TOO) (Vyant Bio, New Jersey, USA) and CancerTYPE ID (Biotheranostics, San Diego, CA, USA) were commonly performed after the failure of the morphological and IHC assessment [9, 10]. The clinical utility of these two assays has been evaluated in few validation studies with an overall sensitivity of 87% to 87.8%, which is favorable to the histopathological method [9, 10].

In our previous study, a 90-gene expression assay was developed to identify 21 common tumor types using RT-PCR methods with total RNA isolated from formalin-fixed, paraffin-embedded (FFPE) tumor tissue [7]. The tumors originated from 21 tissue types, including adrenal gland, brain, breast, cervix, colorectum, endometrium, gastroesophagus, germ cell, head&neck, kidney, liver, lung, melanoma, mesothelioma, neuroendocrine, ovary, pancreas, prostate, sarcoma, thyroid, and urinary system. In a retrospective cohort of 609 clinical specimens, the 90-gene expression assay demonstrated an overall agreement of 90.4% for primary tumors and 89.2% for metastatic tumors. Several studies also demonstrated the excellent performance of the 90-gene expression assay in differentiation diagnosis of triple-negative breast cancer, metastatic brain tumor, squamous cell carcinoma, multiple primary tumors, etc. [11–14]. In the present study, we conducted a large-scale, multicenter study to evaluate the performance of the 90-gene expression assay for tumor tissue of origin identification in real clinical settings.

## Materials and methods

### Ethics statement

The study was conducted under protocols approved by the institutional review boards of each institution, including Beijing Cancer Hospital (BCH, Beijing, China), Fudan University Shanghai Cancer Center (FUSCC, Shanghai, China), and Cancer Hospital of the University of Chinese Academy of Sciences, Zhejiang Cancer Hospital (ZCH, Hangzhou, China). All patients signed informed consent.

### Case selection

In this study, we enrolled a total of 1540 patients between January 2016 and January 2021 from three institutions in China. The inclusion criteria for the multisite study were the following: (1) surgical specimen including primary or metastatic tumors; (2) histologically confirmed tumor type; (3) diagnosis contained within the 21 main tumor types; (4) FFPE tumor specimens processed less than three years from the time of testing; (5) at least 60% tumor cell content available on the hematoxylin and eosin (H&E) stained slide; (6) less than 40% necrosis. Exclusion criteria were (1) tumor specimens obtained after chemotherapy or radiotherapy; (2) cytology cases, biopsy (needle core biopsy [NCB] or fine-needle aspiration [FNA]) cases and decalcified cases. All samples were deidentified, assigned internal accession numbers. The technicians performed the 90-gene expression assay in each institution. Investigators who interpreted the test results were blinded to patients’ medical history, sample location, and histopathological information.

### RNA extraction

For cases meeting the inclusion and exclusion criteria, 5 to 15 5 μm unstained sections were freshly cut for total RNA isolation. The regions of tumor tissue were marked on the H&E-stained slides by senior pathologists at each center (W S and Q Y in BCH, QF W in FUSCC, W W and YY L in ZCH). Tumor cells were then enriched by macro-dissected manually. Total RNA was isolated using FFPE Total RNA Isolation Kit (Canhelp Genomics Co., Ltd, Hangzhou, China) as described before [7]. The concentration and purity of total RNA were measured by spectrophotometer. Exclusion criteria were insufficient RNA (concentration of total RNA, < 60 ng/µl) and low purity (A260/A280 ratio, > 2.1 or < 1.7).

### Gene expression profiling and classification algorithm

The 90-gene expression assay (Canhelp Genomics Co., Ltd) was carried out as previously described [7]. In brief, the reverse transcription was performed on isolated total RNA. Next, the RT-PCR reaction was applied with a 7500 Real Time PCR System (Applied Biosystems) to perform tumor-specific gene expression profiling. The internal control (IC) gene was used to assess the sample quality, while a weak RT-PCR signal (cycle threshold [Ct] value of the IC, greater than 38) was excluded. Additionally, no template control (NTC) was used to evaluate the potential PCR reaction contamination. The sample was excluded when the Ct of the NTC was less than 38.

For each case, the 90-gene classifier analyzed the gene expression pattern of the 90 tumor-specific genes and generated similarity scores for each primary tumor type based on the degree of similarities of the test specimen to the gene expression database. The range of similarity scores was 0 (low similarity) to 100 (high similarity) for each tumor type, and the sum of similarity scores across 21 tumor types was 100.

### Statistical analysis

The internal accession numbers of all cases were finally broken, and test results predicted by the 90-gene expression assay were compared with the reference diagnosis to evaluate the assay performance. As for each tumor type in the panel, sensitivity (or positive percent agreement) was defined as the ratio of true positive results to the total positive samples analyzed. Specificity (or negative percent agreement) was defined as the ratio of true negative results to the total negative samples analyzed. A confusion matrix was generated for each tumor type. All statistical analyses were computed in R software (version 3.6.1). All statistical tests were two-sided, and values of p-value less than 0.05 were considered statistically significant.

## Results

### Patients and specimens

**Fig. 1:**
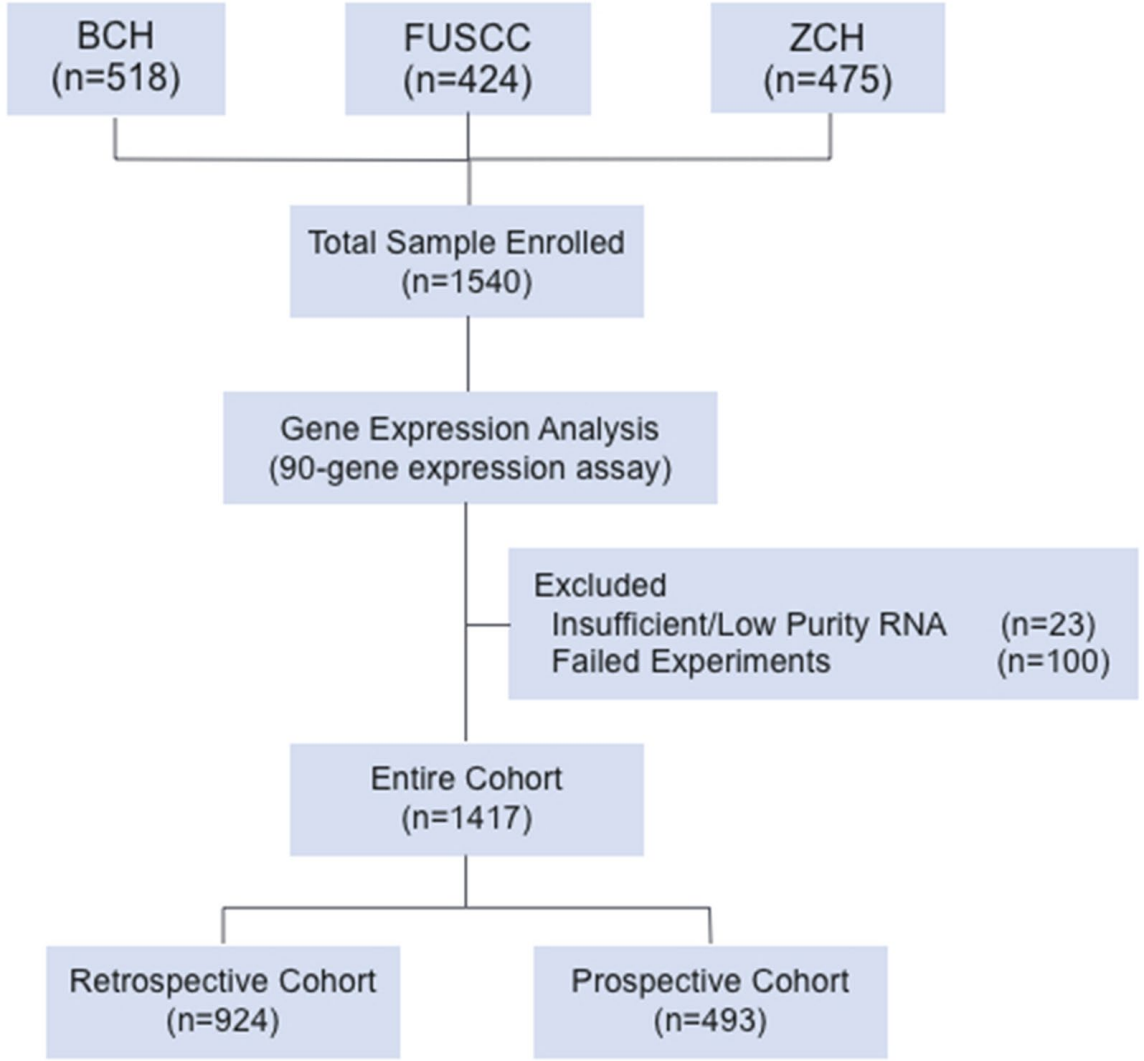
Sample flow diagram

**Fig. 2:**
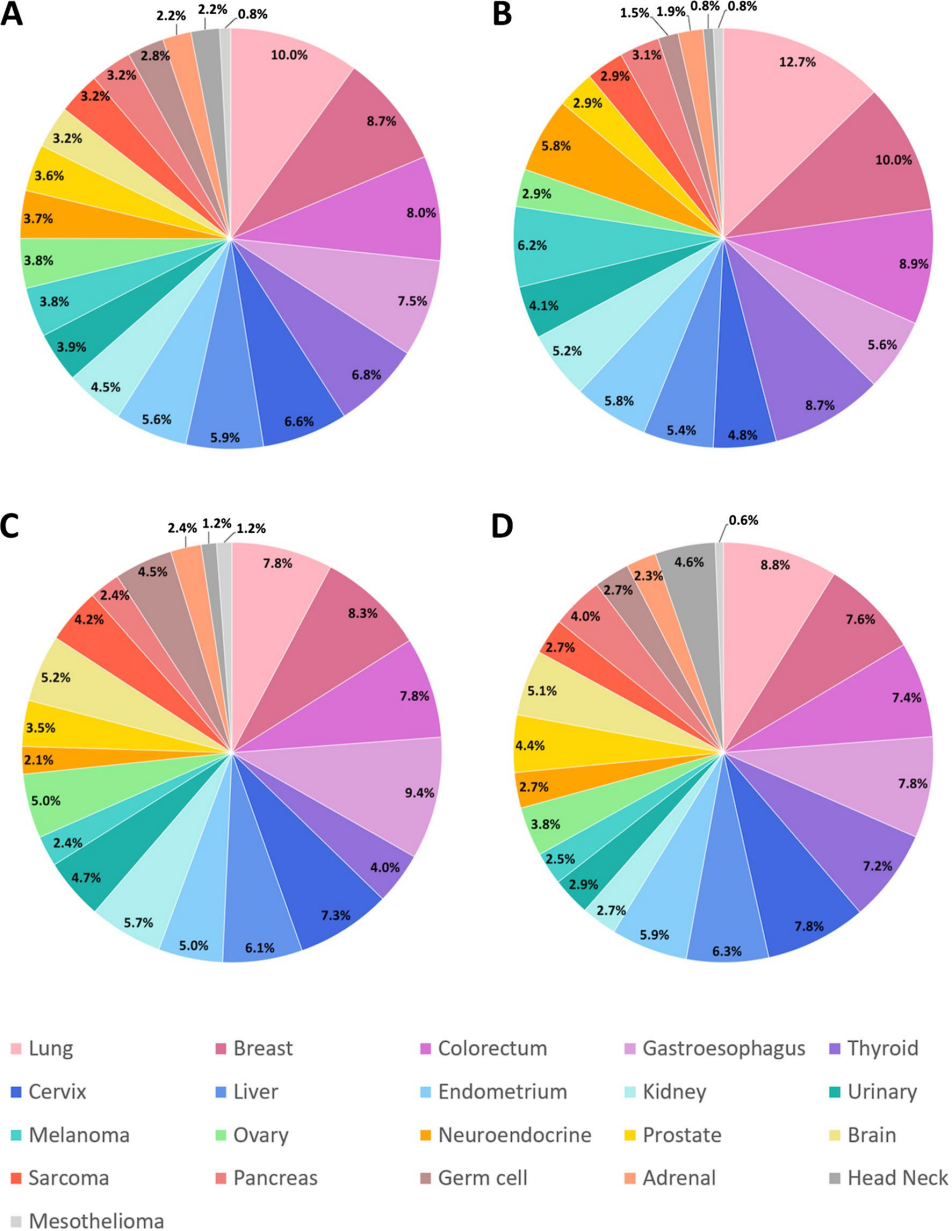
The distribution of tumor types in the **A** entire cohort, **B** Beijing Cancer Hospital, **C** Fudan University Shanghai Cancer Center, and **D** Zhejiang Cancer Hospital

As shown in Fig. 1, 1540 specimens were enrolled from three cancer centers. Among these cases, 23 cases were excluded due to non-sufficient RNA for analysis and/or lower purity, 92 cases had severely degraded nucleic acid, and 8 cases were ruled out due to potential reaction contamination. A total of 1417 samples met all criteria and entered into the study with an overall analytical success rate of 92.0% (1417 of 1540). For details, 924 samples were processed retrospectively during October 2018 and March 2021 (retrospective cohort). In addition, 493 samples with mainly poorly differentiated and undifferentiated tumors were prospectively analyzed from October 2020 to January 2021 in a consecutive manner (prospective cohort). The patients' characteristics according to main tumor types are summarized in Table 1. The median age of the entire patient was 57 years old, ranging from 9 to 88. There were 673 (47.5%) males and 744 (52.5%) females with a sex ratio of 1:1.1. There were 1226 primary tumors and 191 metastatic tumors. As for histological type, the most common type was adenocarcinoma (N = 943, 66.5%), followed by squamous cell carcinoma (N = 166, 11.7%), urothelial carcinoma (N = 55, 3.9%), melanoma (N = 54, 3.8%), neuroendocrine tumor (N = 52, 3.7%), tumor (N = 49, 3.5%), sarcoma (N = 46, 3.2%), germ cell tumor (N = 40, 2.8%) and mesothelioma (N = 12, 0.9%). Of 1417 specimens, the histologic grades of 1112 were assigned, 37.6% (N = 418) were well-moderately differentiated, and 62.4% (N = 694) were poorly differentiated or undifferentiated. The distribution of tumor types in the entire cohort and three institutions were shown in Fig. 2. The most common primary sites included the lung (N = 141, 10.0%), breast (N = 123, 8.7%), colorectum (N = 114, 8.0%), and gastroesophagus (N = 106, 7.5%).

**Table 1:** Patient and tumor demographics and specimen sources

Characteristics	Number of specimens (N = 1417)	Percentage (%)
Study		
Retrospective	924	65.2
Prospective	493	34.8
Centers		
BCH	518	36.6
FUSCC	424	29.9
ZCH	475	33.5
Age (year)		
Median	57	
Range	9–88	
Gender		
Male	673	47.5
Female	744	52.5
Histological type		
Adenocarcinoma	943	66.5
Squamous cell carcinoma	166	11.7
Urothelial carcinoma	55	3.9
Melanoma	54	3.8
Neuroendocrine tumor	52	3.7
Tumor	49	3.5
Sarcoma	46	3.2
Germ cell tumor	40	2.8
Mesothelioma	12	0.9
Histologic grade^a^		
Well-moderately differentiated	418	37.6
Poorly differentiated/Undifferentiated	694	62.4

BCH, Beijing Cancer Hospital; FUSCC, Fudan University Shanghai Cancer Center; ZCH, Zhejiang Cancer Hospital

^a^The differentiation of 305 cases are not defined

### Overall accuracy of the 90-gene expression assay for tumor classification

**Fig. 3:**
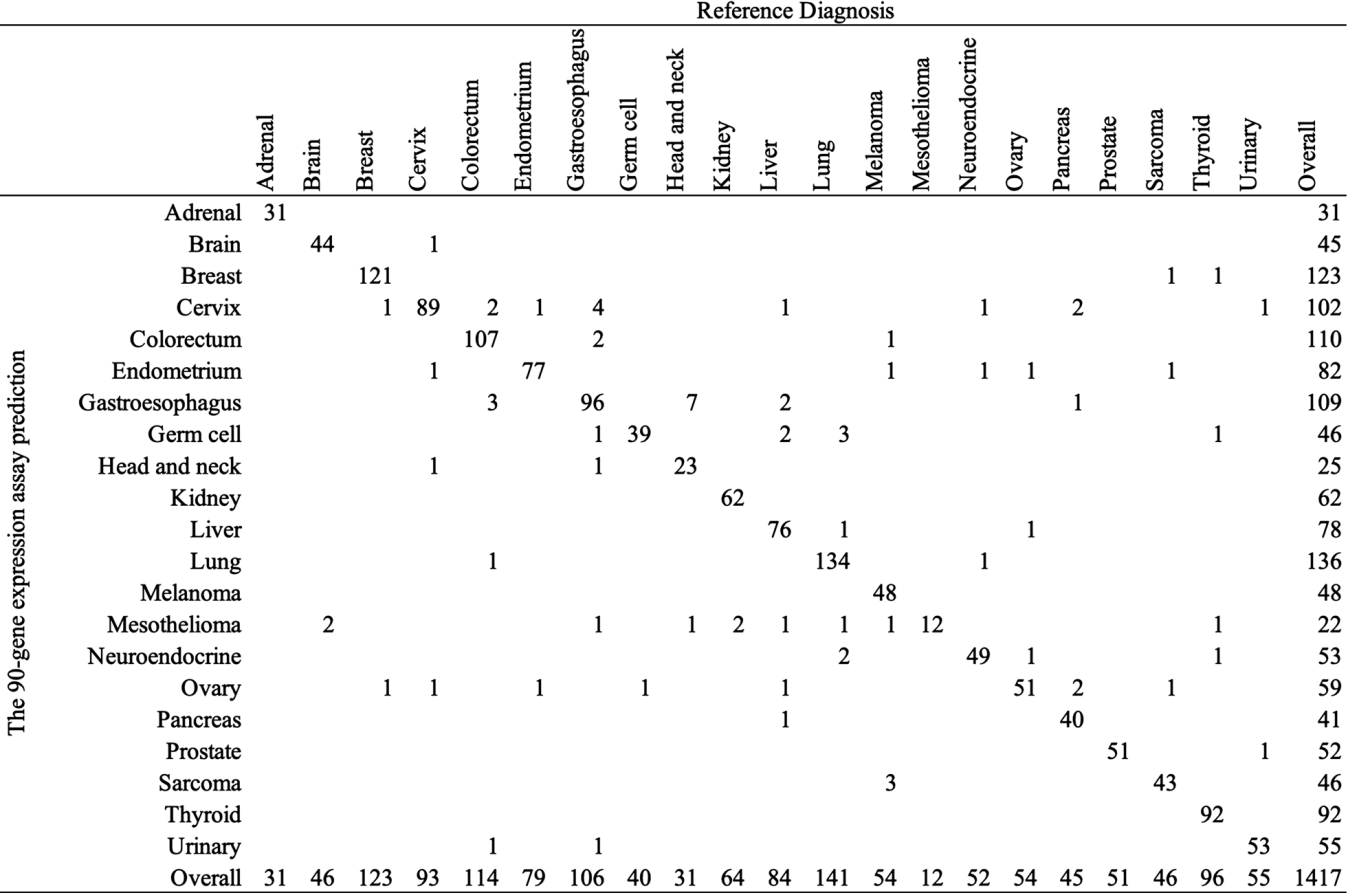
Confusion matrix by tumor type. The reference diagnoses are shown across the top row, and the 90-gene expression assay predictions are shown along the left column

The 90-gene expression assay results showed an overall agreement of 94.4% (1338/1417, 95% CI: 0.93 to 0.96) compared with the pathological diagnosis. The performance of the 90-gene expression assay for each tumor type was shown in Table 2. Of the different tumor types, the sensitivities were ranged from 74.2% (head&neck) to 100% (adrenal, mesothelioma, and prostate). Sensitivities for the most prevalent cancers of lung, breast, colorectum, and gastroesophagus are 95.0%, 98.4%, 93.9%, and 90.6%, respectively. Overall, 18 out of 21 tumor types had sensitivities greater than 90%, and all 21 tumor types had specificities greater than 99%. A confusion matrix of the relationship of predicted results and reference diagnosis was shown in Fig. 3.

**Table 2:** Performance of the 90-gene expression assay in 21 tumor types

Tumor types	Number	Agreement	Sensitivity	Specificity	PPV	NPV
Adrenal	31	31	100.0%	100.0%	100.0%	100.0%
Brain	46	44	95.7%	99.9%	97.8%	99.9%
Breast	123	121	98.4%	99.8%	98.4%	99.8%
Cervix	93	89	95.7%	99.0%	87.3%	99.7%
Colorectum	114	107	93.9%	99.8%	97.3%	99.5%
Endometrium	79	77	97.5%	99.6%	93.9%	99.9%
Gastroesophagus	106	96	90.6%	99.0%	88.1%	99.2%
Germ cell	40	39	97.5%	99.5%	84.8%	99.9%
Head&neck	31	23	74.2%	99.9%	92.0%	99.4%
Kidney	64	62	96.9%	100.0%	100.0%	99.9%
Liver	84	76	90.5%	99.8%	97.4%	99.4%
Lung	141	134	95.0%	99.8%	98.5%	99.5%
Melanoma	54	48	88.9%	100.0%	100.0%	99.6%
Mesothelioma	12	12	100.0%	99.3%	54.5%	100.0%
Neuroendocrine	52	49	94.2%	99.7%	92.5%	99.8%
Ovary	54	51	94.4%	99.4%	86.4%	99.8%
Pancreas	45	40	88.9%	99.9%	97.6%	99.6%
Prostate	51	51	100.0%	99.9%	98.1%	100.0%
Sarcoma	46	43	93.5%	99.8%	93.5%	99.8%
Thyroid	96	92	95.8%	100.0%	100.0%	99.7%
Urinary	55	53	96.4%	99.9%	96.4%	99.9%
Total	1417	1338	Accuracy = 94.4%			

PPV, positive prediction value; NPV, negative prediction value

### Analysis by clinicopathological subsets

The performance of the 90-gene expression assay in clinicopathological subsets was shown in Table 3. In the present study, retrospective cohort (N = 924) and prospective cohort (N = 493) were established to comprehensively evaluate the performance of the 90-gene expression assay in real clinical settings. Accuracy from the prospective cohort was slightly lower than the retrospective cohort (92.1% versus 95.7%, p-value = 0.007). In an analysis comparing well-moderately differentiated and poorly differentiated or undifferentiated tumors, the 90-gene expression assay showed satisfying performance for accurate identification of a primary site, 95.5% (399 of 418) for well-moderately differentiated tumors and 94.5% (656 of 694) for poorly differentiated or undifferentiated tumors, with no statistically different (p-value = 0.59). Among different histological types, agreement rates between the 90-gene expression assay predictions and the reference diagnosis were 95.2% (898 of 943) for adenocarcinoma, 91.0% (151 of 166) for squamous cell carcinoma, 95.4% (53 of 55) for urothelial carcinoma, 88.9% (48 of 54) for melanoma, 94.2% (49 of 52) for neuroendocrine tumor and 91.8% (45 of 49) for tumor, 93.5% (43 of 46) for sarcoma, 97.5% (39 of 40) for germ cell tumor and 100% (12 of 12) for mesothelioma (p-value = 0.23). For the squamous cell carcinomas (N = 166) originated from cervix (N = 89), head&neck (N = 31), gastroesophageal (N = 27), and lung (N = 19), the agreements for tumor classification were 98.9% (88 of 89), 74.2% (23 of 31), 85.2% (23 of 27) and 89.5% (17 of 19), respectively. Of 52 neuroendocrine tumor cases, their tissue of origins was composed of the thyroid (N = 23), pancreas (N = 10), lung (N = 7), cervix (2), skin (N = 1), urinary (N = 1), colorectum (N = 1), and undefined (N = 7). The overall accuracy for neuroendocrine tumors reached 94.2%. In addition, the overall accuracy of the three study sites was 94.4% (489 of 518) at BCH, 95.0% (403 of 424) at FUSCC, and 93.9% (446 of 475) at ZCH. The assay performance across different centers was not statistically different (p-value = 0.75).

**Table 3:** Performance of the 90-gene expression assay in clinicopathological subsets

Clinical variables	Number	Agreement	Accuracy (%)
Study			
Retrospective	924	884	95.7
Prospective	493	454	92.1
Histologic grade			
Well-moderately differentiated	418	399	95.5
Poorly differentiated/Undifferentiated	694	656	94.5
Histological type			
Adenocarcinoma	943	898	95.2
Squamous cell carcinoma	166	151	91.0
Urothelial carcinoma	55	53	95.4
Melanoma	54	48	88.9
Neuroendocrine tumor	52	49	94.2
Tumor	49	45	91.8
Sarcoma	46	43	93.5
Germ cell tumor	40	39	97.5
Mesothelioma	12	12	100
Institution			
BCH	518	489	94.4
FUSCC	424	403	95.0
ZCH	475	446	93.9

### Analysis of discordant specimens

A total of 79 tumor specimens had discordant predictions compared with reference diagnosis. Additional file 1: Table S1 investigated all cases with discordant results of the 90-gene expression assay. The Top-5 common misclassified tumor types were gastroesophagus (N = 10), head&neck (N = 8), liver (N = 8), lung (N = 7), and colorectum (N = 7). Surprisingly, we noticed that eight head&neck tumors were misclassified, among which seven cases were identified as gastroesophageal tumors. The histological types of misclassified specimens included poorly differentiated or undifferentiated (N = 38), well-moderately differentiated tumors (N = 19), and undefined (N = 22).

## Discussion

In the clinic, the identification of tumor type is crucial for optimal treatment selection when a patient diagnosed with a malignant tumor. The traditional diagnosis of tumor type requires a comprehensive analysis of the clinical and pathological findings. Imaging techniques including computed tomography (CT), magnetic resonance imaging (MRI), positron emission tomography-computed tomography (PET-CT) scans are typically used for primary site detection in clinics. However, a recent meta-analysis of PET-CT in 1942 patients from 20 centers found a primary tumor detection rate of 40.9% (39.0% to 42.9%), which is still limited for identifying tumor tissue of origin [15].

In routine pathological diagnostic practice, morphological and IHC assessments were two relatively cost-efficient and no burden methods for patients, which could identify a tumor type in most cases. Nevertheless, the diagnosis of patients with poorly differentiated or undifferentiated tumors is not straightforward because tumors often lack the typical features [16]. Several studies reported an agreement of 69–71% in the characterization of poorly differentiated or undifferentiated carcinomas by performing the IHC and morphology analysis [17, 18].

In the recent decade, studies investigated that distinct tumor types have recognizable differences in gene expression patterns. When tumor metastasis occurs, the gene expression profile of the metastatic foci will maintain the gene expression profile of the primary tumor. Based on this finding, the tumor type of one tumor sample could be elucidated by comparing its gene expression pattern with the gene expression pattern in tumors with known tumor types [19, 20]. Several gene expression assays such as the TOO and CancerTYPE ID have been developed based on mRNA and commercialized to predict the putative primary site for patients with uncertain diagnoses [9, 10]. The TOO test reported by Monzon et al. was a microarray-based test on 1550 genes to differentiate 15 main tumor types. In a blinded validation study that included 547 frozen tumor specimens, the TOO test showed an 87.8% overall agreement with the reference diagnosis [9]. For the CancerTYPE ID assay, Erlander et al. developed a 92-gene real-time PCR assay for identifying the primary site of 28 common tumor types. A multisite validation study used the assay on 790 FFPE tumor specimens and demonstrated an overall sensitivity of 87% in primary site identification [10].

Recently, with the advance of NGS techniques, genomic alterations and DNA methylation have also been applied for tumor molecular classification. Alexander et al. applied machine learning to the assessment of genomic alteration data (468 cancer-associated genes) to predict the tissue of origin, with an overall accuracy of 74.1% in an independent cohort [6]. Sebastian et al. reported a DNA-methylation based test named “EPICUP” for identifying the tissue of origin of CUP. In a CUP validation cohort, EPICUP correctly predicted a primary site in 87% of CUP patients [21]. Moreover, researchers start to investigate the possibility of classifying tumors using less invasive procedures. One exciting approach was explored by M. C. et al., who analyzed the methylation patterns obtained from circulating cell-free DNA (cfDNA) to detect more than 50 cancer types [8]. In a validation cohort of 1354 cases, targeted methylation analysis demonstrated an overall sensitivity of 54.9% and a specificity of > 99%.

This is, to our knowledge, the largest clinical validation study of a gene expression assay for tumor origin identification to date. Overall, the 90-gene expression assay correctly distinguishes tumor type in 94.4% of specimens, which is favorable with the other two commercially available tests (TOO and CancerTYPE ID) with 87%-87.8% accuracy [9, 10]. Furthermore, the present study also established a large-scale prospective cohort (N = 493) to assess the utilization of the 90-gene expression assay in a real clinical setting. Although the accuracy of the prospective cohort (92.1%) was slightly lower than the retrospective cohort (95.7%), it was still superior to the previous studies on tumor classification (87%-87.8%) [7]. Our results show that there is no significant difference in the performance of the gene expression assay for poorly differentiated/undifferentiated and well-moderately differentiated tumors (94.5% versus 95.5%, respectively), suggesting that 90-gene expression patterns of the tumor cells are robust and rarely affected by the loss of cell differentiation.

The present study still had several limitations. The first limitation was the exclusion of suboptimal specimens, such as biopsy samples (NCB or FNA), cytology samples, and samples with excess necrosis or few tumor contents. However, these types of samples are common and usually difficult to diagnosis in clinics. Further verification study is needed to validate the performance of the 90-gene expression assay for suboptimal specimens. In addition, although the 90-gene expression assay achieved overall high classification accuracy cross different tumor types, we found that the performance in identifying the head&neck tumor was not optimal. In this study, eight of 31 head&neck tumors were misidentified, whereas seven of eight misclassified cases were identified as gastroesophageal tumors. Given the conjunction of esophagus and head&neck in anatomy, the mRNA expression, DNA methylation, and somatic copy-number alterations data between esophagus squamous cell carcinoma and head&neck squamous cell carcinoma were demonstrated with a strong resemblance [22]. Gene expression analyses with the 90-gene expression assay also reflect this biologic intersection and provide additional insight into the origin of these tumors. For this instance, additional effort was needed to improve the algorithm performance for distinguishing the head&neck tumors and gastroesophageal tumors. Moreover, the predictions should be interpreted in conjunction with pathological diagnosis and clinical information when the tumor sample was predicted as head&neck and/or gastroesophageal tumors during clinical use.

## Conclusion

These findings showed robust performance of the 90-gene expression assay for identifying the tumor tissue of origin and support the use of molecular testing as an adjunct to tumor classification, especially to those poorly differentiated or undifferentiated tumors in clinical practice.

## Supplementary Information

**Additional file 1: Table S1.** Investigation of cases with discordant results of the 90-gene expression assay.

## Abbreviations

BCH: Beijing Cancer Hospital;
FUSCC: Fudan University Shanghai Cancer Center;
ZCH: Zhejiang Cancer Hospital
