# Supplementary material for: Clinical validation of a 90-gene expression test for tumor tissue of origin diagnosis: a large-scale multicenter study of 1417 patients
Source: J Transl Med. 2022 Mar 7;20:114. doi: 10.1186/s12967-022-03318-6 (PMC8900384; doi:10.1186/s12967-022-03318-6)
Supplement: Supplementary file 1 — Additional file 1: Table S1. Investigation of cases with discordant results of the 90-gene expression assay. [file 12967_2022_3318_MOESM1_ESM.docx]

Additional Material

# Additional Tables

**Additional Table 1.** Investigation of cases with discordant results of the 90-gene expression assay

| **Number** | **Gender** | **Age** | **Reference Diagnosis** | **The 90-gene expression assay results** | **Differentiation** | **Histological type** |
| --- | --- | --- | --- | --- | --- | --- |
| 1 | Female | 45 | Brain | Mesothelioma | Well | Tumor |
| 2 | Male | 34 | Brain | Mesothelioma | Poorly | Tumor |
| 3 | Female | 45 | Breast | Cervix | Well | Adenocarcinoma |
| 4 | Female | 53 | Breast | Ovary | Poorly | Adenocarcinoma |
| 5 | Female | 55 | Cervix | Brain | Poorly | Tumor |
| 6 | Female | 69 | Cervix | Ovary | Poorly | Adenocarcinoma |
| 7 | Female | 58 | Cervix | Head&neck | Poorly | Squamous cell carcinoma |
| 8 | Female | 45 | Cervix | Endometrium | Poorly | Adenocarcinoma |
| 9 | Female | 48 | Colorectum | Cervix | Well | Adenocarcinoma |
| 10 | Male | 59 | Colorectum | Gastroesophagus | Poorly | Adenocarcinoma |
| 11 | Male | 73 | Colorectum | Gastroesophagus | - | Adenocarcinoma |
| 12 | Male | 59 | Colorectum | Gastroesophagus | Poorly | Adenocarcinoma |
| 13 | Female | 60 | Colorectum | Lung | - | Adenocarcinoma |
| 14 | Female | 28 | Colorectum | Cervix | - | Adenocarcinoma |
| 15 | Male | 49 | Colorectum | Urinary | Poorly | Adenocarcinoma |
| 16 | Female | 62 | Endometrium | Cervix | Poorly | Adenocarcinoma |
| 17 | Female | 55 | Endometrium | Ovary | - | Adenocarcinoma |
| 18 | Female | 57 | Gastroesophagus | Cervix | Well | Adenocarcinoma |
| 19 | Female | 73 | Gastroesophagus | Cervix | Poorly | Adenocarcinoma |
| 20 | Female | 60 | Gastroesophagus | Colorectum | Well | Adenocarcinoma |
| 21 | Female | 61 | Gastroesophagus | Colorectum | Poorly | Adenocarcinoma |
| 22 | Male | 54 | Gastroesophagus | Head&neck | - | Squamous cell carcinoma |
| 23 | Female | 64 | Gastroesophagus | Cervix | Well | Squamous cell carcinoma |
| 24 | Male | 71 | Gastroesophagus | Germ cell | Well | Adenocarcinoma |
| 25 | Male | 55 | Gastroesophagus | Mesothelioma | Well | Squamous cell carcinoma |
| 26 | Female | 60 | Gastroesophagus | Cervix | Poorly | Adenocarcinoma |
| 27 | Male | 65 | Gastroesophagus | Urinary | Well | Squamous cell carcinoma |
| 28 | Female | 17 | Germ cell | Ovary | - | Germ cell tumor |
| 29 | Male | 63 | Head&neck | Gastroesophagus | Poorly | Squamous cell carcinoma |
| 30 | Male | 69 | Head&neck | Gastroesophagus | Poorly | Squamous cell carcinoma |
| 31 | Male | 34 | Head&neck | Gastroesophagus | Well | Squamous cell carcinoma |
| 32 | Male | 64 | Head&neck | Gastroesophagus | Well | Squamous cell carcinoma |
| 33 | Male | 78 | Head&neck | Gastroesophagus | Poorly | Squamous cell carcinoma |
| 34 | Male | 54 | Head&neck | Gastroesophagus | Well | Squamous cell carcinoma |
| 35 | Male | 63 | Head&neck | Gastroesophagus | - | Squamous cell carcinoma |
| 36 | Male | 62 | Head&neck | Mesothelioma | - | Squamous cell carcinoma |
| 37 | Male | 73 | Kidney | Mesothelioma | Well | Adenocarcinoma |
| 38 | Male | 63 | Kidney | Mesothelioma | Well | Adenocarcinoma |
| 39 | Female | 48 | Liver | Ovary | Poorly | Adenocarcinoma |
| 40 | Male | 82 | Liver | Germ cell | Poorly | Adenocarcinoma |
| 41 | Female | 65 | Liver | Cervix | Poorly | Adenocarcinoma |
| 42 | Male | 53 | Liver | Germ cell | Poorly | Adenocarcinoma |
| 43 | Female | 68 | Liver | Pancreas | Well | Adenocarcinoma |
| 44 | Male | 69 | Liver | Gastroesophagus | Poorly | Adenocarcinoma |
| 45 | Male | 62 | Liver | Mesothelioma | Well | Adenocarcinoma |
| 46 | Female | 48 | Liver | Gastroesophagus | Poorly | Adenocarcinoma |
| 47 | Female | 60 | Lung | Germ cell | - | Adenocarcinoma |
| 48 | Male | 60 | Lung | Liver | Poorly | Squamous cell carcinoma |
| 49 | Female | 58 | Lung | Germ cell | Well | Adenocarcinoma |
| 50 | Male | 65 | Lung | Neuroendocrine | Poorly | Adenocarcinoma |
| 51 | Male | 70 | Lung | Neuroendocrine | Poorly | Adenocarcinoma |
| 52 | Male | 67 | Lung | Mesothelioma | Well | Squamous cell carcinoma |
| 53 | Male | 57 | Lung | Germ cell | Poorly | Tumor |
| 54 | Female | 44 | Melanoma | Sarcoma | - | Melanoma |
| 55 | Female | 39 | Melanoma | Sarcoma | - | Melanoma |
| 56 | Male | 71 | Melanoma | Mesothelioma | - | Melanoma |
| 57 | Female | 57 | Melanoma | Colorectum | - | Melanoma |
| 58 | Female | 53 | Melanoma | Endometrium | - | Melanoma |
| 59 | Female | 27 | Melanoma | Sarcoma | - | Melanoma |
| 60 | Male | 66 | Neuroendocrine | Lung | - | Neuroendocrine tumor |
| 61 | Female | 46 | Neuroendocrine | Cervix | Poorly | Neuroendocrine tumor |
| 62 | Female | 35 | Neuroendocrine | Endometrium | Poorly | Neuroendocrine tumor |
| 63 | Female | 49 | Ovary | Neuroendocrine | Poorly | Adenocarcinoma |
| 64 | Female | 68 | Ovary | Liver | Poorly | Adenocarcinoma |
| 65 | Female | 59 | Ovary | Endometrium | Poorly | Adenocarcinoma |
| 66 | Female | 66 | Pancreas | Ovary | Poorly | Adenocarcinoma |
| 67 | Female | 46 | Pancreas | Cervix | Poorly | Adenocarcinoma |
| 68 | Female | 49 | Pancreas | Gastroesophagus | Poorly | Adenocarcinoma |
| 69 | Female | 63 | Pancreas | Ovary | Poorly | Adenocarcinoma |
| 70 | Female | 56 | Pancreas | Cervix | Poorly | Adenocarcinoma |
| 71 | Female | 43 | Sarcoma | Endometrium | - | Sarcoma |
| 72 | Female | 49 | Sarcoma | Ovary | - | Sarcoma |
| 73 | Female | 33 | Sarcoma | Breast | Well | Sarcoma |
| 74 | Female | 31 | Thyroid | Neuroendocrine | - | Adenocarcinoma |
| 75 | Female | 33 | Thyroid | Mesothelioma | - | Adenocarcinoma |
| 76 | Female | 27 | Thyroid | Breast | - | Adenocarcinoma |
| 77 | Male | 38 | Thyroid | Germ cell | - | Adenocarcinoma |
| 78 | Male | 65 | Urinary | Prostate | Poorly | Urothelial carcinoma |
| 79 | Female | 41 | Urinary | Cervix | Poorly | Urothelial carcinoma |
